# Lower-limb asymmetries in jump athletes during single-leg drop jump

**DOI:** 10.3389/fphys.2025.1702161

**Published:** 2025-11-13

**Authors:** Yunman Li, Xinxin Zhang, Yong Zhou, Yuliang Sun

**Affiliations:** 1 School of Physical Education, Shaanxi Normal University, Xi’an, China; 2 College of physical education and health, Guilin Institute of Information Technology, Guilin, China

**Keywords:** lower limb extremity, biomechanics, athletic performance, drop jump, asymmetry

## Abstract

**Background:**

This study investigated lower-limb biomechanical asymmetries during single-leg drop jumps (SLDJ) in elite male long and high jumpers.

**Methods:**

Twenty athletes performed SLDJ from 30-cm and 40-cm heights using dominant and non-dominant legs. Three-dimensional kinematic and kinetic data were collected using a motion capture system and force platforms. Measured variables included joint angles, moments, peak vertical ground reaction force (Peak vGRF), loading rate, reactive strength index (RSI), and absolute symmetry index (ASI%). Data were analyzed using a two-way repeated-measures ANOVA.

**Results:**

The dominant leg exhibited significantly greater ankle range of motion (*p* < 0.05), hip joint moment (*p* < 0.05), ankle joint moment (*p* < 0.001), and peak vGRF (*p* < 0.001) compared to the non-dominant leg. Furthermore, knee joint moments (*p* < 0.05) and RSI (*p* < 0.001) were significantly greater at the 40 cm height than at the 30 cm height. The ASI% for the peak vGRF (30 cm: 10.74% ± 9.24%, 40 cm: 14.87% ± 13.75%) and the loading rate (30 cm: 15.47% ± 14.81%, 40 cm: 20.27% ± 9.80%) exceeded 10%, which indicated asymmetry between the two legs during the single-leg drop jump impact.

**Conclusion:**

These findings suggest a trend wherein inter-limb asymmetry during the single-leg drop jump appeared to become more pronounced with increasing drop height. This observation may offer valuable insight for sport-specific performance assessment and targeted injury prevention.

## 1 Introduction

Lower limb asymmetry is defined as measurable bilateral differences in function or performance (Sun et al., 2025). It is typically characterized by differences between limbs in strength, explosive power, and range of motion (Bishop et al., 2016; Willwacher et al., 2017). In competitive sports, such asymmetry arises from sport-specific demands—such as repeated unilateral kicking in soccer (Bishop et al., 2021), directional changes in basketball (Mainer-Pardos et al., 2024), and accumulated single-leg takeoff effects in long jump (dos Santos Silva et al., 2023). In addition, when the degree of asymmetry exceeds 10%, the risk of lower-limb injury increases approximately fourfold (Gustavsson et al., 2006; Pardos-Mainer et al., 2020). In rehabilitation contexts, an asymmetry level below 10% is often considered a reference standard for satisfactory functional recovery (Kyritsis et al., 2016), indicating that the effects of such asymmetries on performance should be interpreted from multiple perspectives (Bishop et al., 2016).

Jumping movements typically have four phases: approach, takeoff, flight, and landing (Hay, 1986). From a biomechanical perspective, athletes often rely on a unilateral takeoff strategy during training and competition (Hay, 1993), which may easily lead to side-to-side differences in lower limb muscle strength, stability, and flexibility (Bishop et al., 2016; Moreno-Villanueva et al., 2025). Consequently, accurately detecting and quantifying these asymmetries has become a central focus in biomechanics research (Bishop et al., 2016). Current assessment methods are generally categorized into bilateral tests (e.g., back squat (Newton et al., 2006; Flanagan and Salem, 2007; Hodges et al., 2011; Sato and Heise, 2012), countermovement jump (Impellizzeri et al., 2007; Yoshioka et al., 2010; Bell et al., 2014), drop jump (Bishop et al., 2019; Lim et al., 2020) and unilateral tests (e.g., single-leg countermovement jump (Jones and Bampouras, 2010; Keeley et al., 2011; Ceroni et al., 2012), single-leg hop (Barber et al., 1990; Noyes et al., 1991; Myers et al., 2014)), with key metrics including jump height, distance, and reactive strength index (RSI) (Bishop et al., 2019). However, traditional bilateral tests may not fully capture the biomechanical demands of sport-specific movements. In contrast, the SLDJ test not only closely replicates the technical features of real-world sport movements (Ross et al., 2005; Richardson et al., 2020) but also effectively activates the stretch-shortening cycle (SSC) mechanism (Bobbert et al., 1987a). It is particularly advantageous in identifying inter-limb asymmetries and functional deficits that bilateral tests may overlook (Huurnink et al., 2019; Lem et al., 2022). Notably, in SLDJ testing, different drop heights significantly alter the mechanical load imposed on muscles, joints, and connective tissues (Hollville et al., 2019), which may further influence the expression of asymmetry in the lower limbs. Despite growing attention to inter-limb asymmetries, most existing studies have concentrated on anterior cruciate ligament (ACL) injury risk and postoperative recovery (Kotsifaki et al., 2023; Shibata et al., 2023), with relatively little emphasis on how such asymmetries manifest in jumping athletes under varying drop heights—a topic that remains underexplored.

Therefore, this study aims to quantify biomechanical asymmetries between the dominant and non-dominant legs in male jump athletes during single-leg drop jumps at different heights. Specifically, we will examine limb-specific differences in kinematic and kinetic parameters, joint work distribution at the hip, knee, and ankle, peak ground reaction forces (vGRF), and symmetry indices. We hypothesize that the dominant leg would exhibit greater hip and ankle joint moments, higher reactive strength index (RSI), and greater peak ground reaction forces (vGRF) compared with the non-dominant leg, and that these inter-limb differences would be more pronounced at the higher drop height.

## 2 Materials and methods

### 2.1 Participants

Twenty elite male jump athletes from the College of Physical Education at Shaanxi Normal University participated in this study. All participants were certified as Chinese National Grade II Athletes, including ten high jumpers and ten long jumpers (age = 20.41 ± 1.11 years; height = 183.17 ± 5.14 cm; body mass = 71.28 ± 4.18 kg). *A priori* power analysis using G*Power 3.1 (effect size f = 0.30, α = 0.05, power = 0.80) indicated a required sample size of 17 participants (Cohen, 1988; Alanazi et al., 2021; Yi et al., 2024). Considering the sample sizes commonly reported in previous single-leg drop jump studies (Walsh et al., 2004; Lem et al., 2022; Pilanthananond et al., 2023) and to ensure adequate statistical power, we recruited 20 athletes to enhance the robustness of our findings. Participants had an average of 7.8 ± 2.3 years of competitive experience and trained at least five times weekly (≥2 h per session), with no lower limb injuries in the preceding 6 months. Before formal testing, baseline assessments of anthropometrics and single-leg drop jump performance were conducted. Independent-samples t-tests revealed no significant differences between high jumpers and long jumpers in any of these baseline measures (all *p* > 0.05), thus justifying their treatment as a homogeneous elite population for subsequent analysis (Table 1). All provided written informed consent, and the study was approved by the Ethics Committee of Shaanxi Normal University (202416044) per the Declaration of Helsinki.

**TABLE 1:** Anthropometrics and Single-leg drop jump height in High Jump and Long Jump Athletes.

Parameter	High jump	Long jump	t	*p*
Height (cm)	183.166 ± 6.203	183.173 ± 4.158	0.003	>0.05
Body mass (kg)	72.570 ± 3.705	69.993 ± 4.411	1.415	>0.05
Age (years)	20.715 ± 1.256	20.108 ± 0.904	1.241	>0.05
Baseline SLDJ height (DL)(cm)	0.193 ± 0.056	0.199 ± 0.062	0.228	>0.05
Baseline SLDJ height (NDL)(cm)	0.191 ± 0.025	0.190 ± 0.027	0.120	>0.05

NDL: Non-dominant leg; DL: dominant leg; SLDJ: Single-leg drop jump.

### 2.2 Apparatus and measurement

This study used ten infrared motion capture cameras (Oqus700+, Qualisys AB, Sweden, 200 Hz) and two force plates (Model 9260AA6, Kistler Instrument, Switzerland, 1000 Hz) to synchronously collect biomechanical data of the hip, knee, and ankle joints during single-leg drop jump tasks performed from 30 cm to 40 cm platforms. The selection of the drop height is primarily based on the findings of previous studies (Wang and Peng, 2014).

**FIGURE 1:**
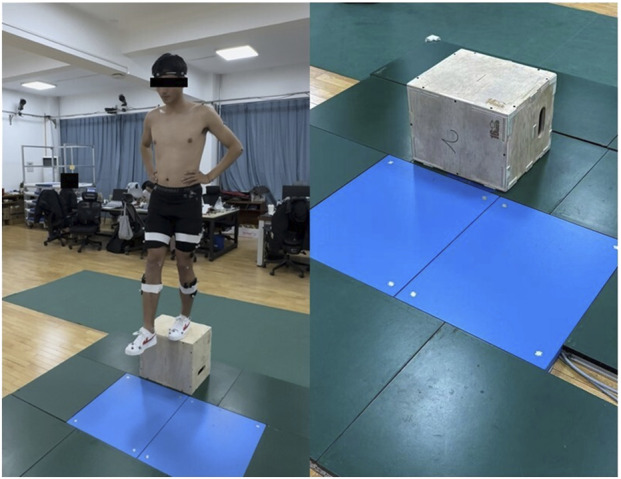
Experimental environment.

Before formal testing, participants first identified their dominant leg by performing a ball-kicking task—the leg used to kick was defined as the dominant leg, while the supporting leg was classified as the non-dominant leg (Edwards et al., 2012; Pappas and Carpes, 2012). For all jump athletes in this study, the kicking leg corresponded to their takeoff leg used during training and competition. Thereafter, all participants completed a standardized warm-up consisting of 5 min of treadmill running at 6.5 km/h followed by dynamic stretching, after which reflective markers were placed according to the calibrated anatomical systems technique (CAST), with 57 markers attached to anatomical landmarks on the upper limbs, trunk, pelvis, and lower limbs, and four rigid marker clusters fixed to the mid-lateral regions of the thighs and shanks bilaterally (Cappozzo et al., 1995); Participants then performed SLDJ from 30 cm to 40 cm heights using both legs, stepping off the platform and immediately jumping upward upon landing with maximal effort (Bobbert et al., 1987a; 1987b). A trial was considered successful only if: (1) the participant maintained hands on the waist throughout the entire movement; (2) the entire foot of the testing leg landed squarely within a central 5-cm tolerance zone of the force plate; and (3) no loss of balance or extra steps occurred after the final landing, with a stable position held until instructed to step off (Ambegaonkar et al., 2011; Tamura et al., 2016). The selection of 30 cm and 40 cm drop heights was guided by previous research (Wang and Peng, 2014). Before data collection, participants practiced to familiarize themselves with the protocol. During the experiment, an average of approximately 3.5 trials were attempted per condition per participant, with an 85% success rate. A 1-min rest was provided between trials. The mean of the three valid trials per condition was used for subsequent analysis to minimize intra-session variability (Figure 1).

### 2.3 Data analysis

In this study, drop jump phases were determined based on both kinetic and kinematic criteria. Initial ground contact was identified when the peak vGRF exceeded 10 N. The ground contact phase was then divided into two segments to distinguish muscle action patterns: the braking phase, from initial contact to maximum knee flexion, representing eccentric loading; and the push-off phase, from maximum knee flexion to toe-off, representing concentric propulsion (Olaf et al., 2013).

Visual 3D biomechanical analysis software (v5, C-Motion, Inc., Germantown, MD, United States) was used to compute the 3D kinematic and kinetic variables of both sides of the lower extremities in the single-leg drop jump. Segmental rotations were described using an X–Y–Z Cardan sequence (right-hand rule) (Lu et al., 2024). Kinematic and kinetic signals were both low-pass filtered with a fourth-order Butterworth filter, using cutoff frequencies of 14 Hz and 50 Hz, respectively (Sun et al., 2015). Hip flexion, knee extension, and ankle dorsiflexion are positive values (+), and the corresponding hip extension, knee flexion, and ankle plantar flexion are negative values (−).

The main kinematic variables included the following: (1) Joint angles of the hip, knee, and ankle in the sagittal plane and ranges of motion (ROM); (2) Joint moment; (3) Drop jump height and contact time; (4) Reactive Strength Index (RSI) = $\frac{jump\;height}{\text{contact}\;\text{time}}$ (Prieske et al., 2019).

In addition, the main kinetic variables included the following: (1) Peak vertical GRF, which was normalized to body mass; (2) The GRF loading rate, which was calculated from the maximum GRF value and time to the maximum GRF; (3) Normalized joint moments; (4) Joint work generation was calculated as the net joint power integrated over time in regions with positive internal power, and work absorption in regions with negative internal power. The contribution of each joint was determined as a percentage of the sum of all three lower-limb joints during each phase (Kotsifaki et al., 2022).

Meanwhile, the absolute symmetry index (ASI) was used to analyse the landing impact symmetry between the dominant and non-dominant legs during the single-leg drop jump (Herzog et al., 1989; Bishop et al., 2016; Björklund et al., 2017), with a focus on the peak vertical ground reaction force (vGRF) and the loading rate, as these are key kinetic parameters for assessing impact loads and injury risk (Wang and Fu, 2019).

$$ASI\left(\%\right)=\frac{\left(D-N\right)}{0.5\times \left(D+N\right)}\times 100$$

where D = dominant leg, N = non-dominant leg; ASI <10% indicates acceptable symmetry (Bosch and Rosenbaum, 2010; Wang and Fu, 2019).

### 2.4 Statistical analysis

All statistical analyses were performed using SPSS Statistics (version 27.0, IBM Corporation, United States). Independent-samples t-tests confirmed no significant baseline differences between high-jump and long-jump athletes in anthropometric characteristics and single-leg drop jump height (all *p* > 0.05), thus justifying the treatment of all participants as a homogeneous elite jumping-athlete group for subsequent analyses. A two-way repeated-measures ANOVA examined the main and interaction effects of leg dominance (dominant vs. non-dominant) and drop height (30 cm vs. 40 cm). The assumption of sphericity was tested using Mauchly’s test, and when violated, the Greenhouse–Geisser correction was applied to adjust the degrees of freedom. Continuous variables are reported as means ± standard deviations (Mean ± SD). The Shapiro–Wilk test was used to assess the normality of the distribution for each variable across both leg conditions before statistical testing. For significant interactions, simple-effects analyses were performed using the Bonferroni method. All tests were two-tailed, with the significance level set at α = 0.05. Only statistically significant p-values (*p* < 0.05) are reported.

## 3 Results

### 3.1 Kinematics and kinetics

The complete dataset is available in Tables 2–4. Analysis revealed no significant differences in lower-limb joint angle variables were observed between the dominant and non-dominant legs across the different drop heights at initial foot contact. For joint ROM, a significant main effect of leg dominance was found for the ankle [F (1, 20) = 8.062, *p* = 0.010, η^2^p = 0.288], with the dominant leg demonstrating significantly greater ROM than the non-dominant leg (*p* < 0.05). Analysis of knee joint ROM revealed a significant leg dominance × height interaction [F (1, 20) = 11.112, *p* = 0.003, η^2^p = 0.369], showing that for the dominant leg, knee joint ROM was significantly greater at 40 cm than at 30 cm (*p* < 0.05).

**TABLE 2:** Summary of two-way repeated measures ANOVA.

Parameters	Height effect		Leg dominance effect		Interaction effect	
	F	*p*	F	*p*	F	*p*
Hip flexion angle (°)	0.654	0.431	1.223	0.443	0.093	0.764
Knee flexion angle (°)	0.488	0.495	0.570	0.460	1.112	0.053
Ankle dorsiflexion angle (°)	0.027	0.871	4.091	0.061	3.432	0.084
Hip ROM (°)	0.174	0.681	3.365	0.083	0.088	0.770
Knee ROM (°)	0.170	0.685	0.045	0.835	11.112	0.003
Ankle ROM (°)	0.639	0.434	8.062	0.010	0.280	0.603
Peak hip moment (Nm/kg)	1.705	0.209	5.363	0.033	0.597	0.450
Peak knee moment (Nm/kg)	6.204	0.023	1.486	0.239	0.051	0.825
Peak ankle moment (Nm/kg)	0.083	0.777	17.278	<0.001	0.227	0.640
RSI (m/s)	6.595	0.019	1.449	0.244	0.009	0.952
Contact time (s)	2.108	0.163	0.236	0.633	0.209	0.653
Jump height (cm)	0.397	0.536	0.310	0.584	1.247	0.278
Peak vGRF (BW)	1.170	0.293	31.456	<0.001	1.063	0.315
Loading rate (BW/s)	4.092	0.057	2.605	0.123	0.354	0.559
Time to peak vGRF (ms)	3.088	0.095	0.451	0.510	0.863	0.365

**TABLE 3:** Post Hoc multiple comparisons for main effects.

Parameters	Height		*p*	Leg dominance		*p*
	30 cm	40 cm		NDL	DL	
Ankle ROM (°)	46.294 ± 8.185	47.106 ± 5.036	0.434	45.243 ± 7.529	48.157 ± 6.175*	0.010
Peak hip moment (Nm/kg)	−5.749 ± 2.159	−6.664 ± 4.410	0.209	−5.334 ± 2.726	−7.080 ± 4.217*	0.033
Peak knee moment (Nm/kg)	5.464 ± 2.276	6.857 ± 3.653*	0.023	5.839 ± 2.710	6.481 ± 3.234	0.239
Peak ankle moment (Nm/kg)	−3.149 ± 0.679	−3.093 ± 0.942	0.777	−2.723 ± 0.690	−3.519 ± 1.090***	<0.001
RSI (m/s)	0.601 ± 0.254	0.717 ± 0.209*	0.019	0.685 ± 0.196	0.632 ± 0.253	0.244
Peak vGRF (BW)	4.199 ± 0.653	4.407 ± 1.083	0.293	3.901 ± 0.862	4.704 ± 1.084***	<0.001

**TABLE 4:** Post Hoc multiple comparisons for interaction effects.

Parameter	30 cm-NDL	40 cm-NDL	*p*	30 cm-DL	40 cm-DL	*p*
Knee ROM (°)	48.661 ± 1.970	44.613 ± 2.304	0.157	46.567 ± 2.057	48.531 ± 1.924*	0.024

*p < 0.05; **p < 0.001; NDL: Non-dominant leg; DL: dominant leg; ROM: joint range of motion; RSI: reactive strength index; Peak vGRF: peak vertical ground reaction force.

Peak joint moment analysis revealed that hip moments exhibited significant main effects of leg dominance [F (1, 20) = 5.363, *p* = 0.033, η^2^p = 0.240], where the dominant leg generated significantly greater moments than the non-dominant leg (*p* < 0.05). Knee moments showed a significant main effect of landing height [F (1, 20) = 6.204, *p* = 0.023, η^2^p = 0.25], with significantly greater moments at the 30 cm height than at the 40 cm height (*p* < 0.05). Ankle moments displayed a significant main effect of leg dominance [F (1, 20) = 17.278, *p* < 0.001, η^2^p = 0.504], where the dominant leg generated significantly greater moments than the non-dominant leg (*p* < 0.05).

Additionally, although contact time and jump height did not differ significantly, their mean ± SD values were: contact time—30 cm: non-dominant leg 0.293 ± 0.037 s, dominant leg 0.278 ± 0.048 s; 40 cm: non-dominant leg 0.294 ± 0.046 s, dominant leg 0.284 ± 0.063 s; jump height—30 cm: non-dominant leg 0.183 ± 0.048 m, dominant leg 0.200 ± 0.061 m; 40 cm: non-dominant leg 0.195 ± 0.058 m, dominant leg 0.196 ± 0.051 m. In contrast, the reactive strength index (RSI) showed a significant main effect of landing height [F (1, 20) = 6.595, *p* = 0.019, η^2^p = 0.258], with significantly greater RSI values at the 40 cm height compared to the 30 cm height (*p* < 0.05). Peak vGRF analysis revealed a significant main effect of leg dominance [F (1, 20) = 31.456, *p* < 0.001, η^2^p = 0.623], with the dominant leg demonstrating significantly greater peak vGRF than the non-dominant leg (*p* < 0.001). However, no significant differences existed between legs in the time to peak vGRF or the GRF loading rate.

### 3.2 Symmetry

**FIGURE 2:**
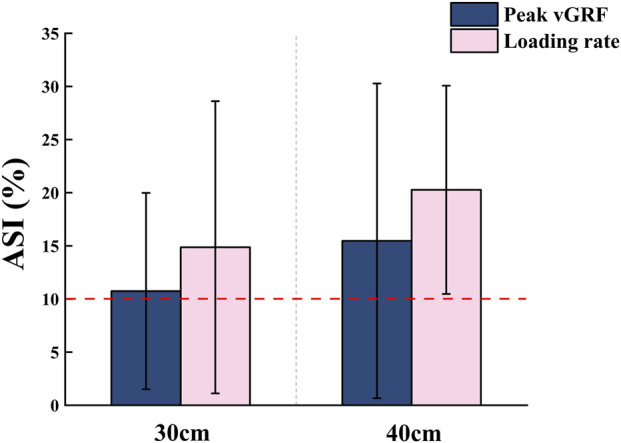
Absolute symmetry index (ASI) for the peak vGRF and loading rate during single-leg drop jump.

The ASI for peak vGRF was 10.74% ± 9.24% at the 30 cm drop height and 14.87% ± 13.75% at the 40 cm drop height (a difference of 4.13 percentage points). For loading rate, the ASI was 15.47% ± 14.81% at 30 cm and 20.27% ± 9.80% at 40 cm (a difference of 4.80 percentage points) (Figure 2).

### 3.3 Joint work and contribution

**FIGURE 3:**
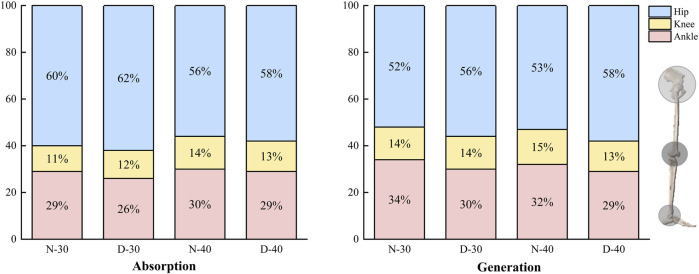
The average percentage of work contributions from the hip, knee, and ankle joints during the absorption and generation phases of the single-leg drop jump is 30 cm and 40 cm, respectively.

During the absorption phase of the single-leg drop jump, the dominant leg exhibited a redistribution of joint work compared to the non-dominant leg, characterized by an increased hip contribution (from 60% to 62%), a decreased ankle contribution (from 29% to 26%), and minimal change in knee work. A similar pattern was observed during the generation phase, where hip contribution increased from 52% to 56% and ankle contribution decreased from 34% to 30%, with knee work remaining similar or slightly lower. These consistent redistribution patterns across both 30 cm and 40 cm drop heights indicate that the direction of these leg-specific strategies was not altered by drop height (Figure 3).

## 4 Discussion

This study systematically analyzed lower limb biomechanics in jumping athletes during single-leg drop jumps from different heights, focusing on the relationship between inter-limb asymmetry and drop height. The results revealed significant kinetic differences between limbs and across heights. A proximal compensation strategy was observed in joint work distribution, characterized by greater contributions from the hip and ankle joints with relatively reduced knee involvement. Furthermore, the asymmetry index for peak vertical ground reaction force and average loading rate exceeded the 10% threshold, with values increasing at higher drop heights. These findings suggest the presence of inter-limb asymmetry during single-leg drop jumps. The observed trend of height-dependent exacerbation appears to align with the study’s initial hypothesis.

### 4.1 Joint kinematics and kinetics

Examination of sagittal-plane knee ROM during the landing phase revealed significant differences between the dominant and non-dominant legs and across drop heights. These findings mirror those of a previous study (Wang and Fu, 2019). However, joint angles at initial contact showed no statistical differences, suggesting that athletes adopted a similar landing posture regardless of leg dominance.

Our data demonstrated significant leg dominance effects at both the hip and ankle, with the dominant legs consistently producing higher peak moments, a pattern consistent with Ren et al. (2025) in isokinetic strength assessments. Although the knee showed a similar trend, the interlimb difference was not statistically significant despite peak moments increasing with drop height, which aligns with Nelson et al. (2018) reporting minimal interlimb differences in knee joint kinetics during landing in healthy individuals. Collectively, these findings support the proximal-to-distal torque gradient described by Dufek and Bates (1991), whereby proximal joints, especially the hip, bear greater mechanical demands during vertical impacts. This joint-specific loading strategy and pronounced hip and ankle dominance underscore the importance of systematically monitoring asymmetries in both proximal and distal joints to optimise force transmission, enhance performance, and mitigate injury risk in unilateral jumping tasks. Furthermore, the limb dominance observed at the hip and ankle in our male collegiate jump athletes parallels the limb- and sex-based joint moment differences reported by Decker et al. (2003), and likely reflects long-term unilateral loading adaptations to the mechanical demands of high jump and long jump (Hay, 1993). Practically, this pattern suggests that strengthening the non-dominant hip and ankle through targeted unilateral eccentric and plyometric training may help restore kinetic balance and reduce injury susceptibility.

Similarly, while neither ground contact time nor flight height differed significantly between legs, mean values consistently favoured the dominant leg, in agreement with earlier research (Kuromaru et al., 2025). Finally, athletes require longer ground contact times to dissipate impact as drop height increases, thereby lengthening ground contact time. Since plyometric jump training (PJT) enhances reactive strength index (RSI) through the stretch-shortening cycle (SSC) mechanism—with jump height serving as a direct indicator of SSC efficiency—improvements in reactive strength index are often accompanied by increases in jump height, a conclusion supported by both previous studies and our own findings (Ramirez-Campillo et al., 2023). These findings imply that plyometric training intensity and drop height should be progressively individualized to prevent excessive eccentric stress and asymmetry exacerbation.

### 4.2 Symmetry

Peak vGRF analysis in our study showed that male jump athletes consistently produced greater impact forces with their dominant leg during single-leg landings. However, no interlimb differences were observed in the timing-related variables, such as time to peak vGRF or loading rate. This finding contrasts with previous studies on bilateral landings in female athletes, where peak vGRF was generally distributed symmetrically between legs (Sinsurin et al., 2017). Such symmetry has been attributed to a centrally coordinated shock attenuation strategy (Aizawa et al., 2018). In contrast, our results suggest that trained male jumpers may exhibit a force-dominant leg bias under unilateral conditions without changes in the temporal aspects of impact absorption. These findings align with prior reports indicating that sex, task type, and sport-specific loading histories can influence asymmetry patterns during landing (Maloney, 2019).

Based on the findings of this study, the asymmetry indices for both peak ground reaction force and loading rate exceeded the conventional 10% threshold for balanced loading, with the asymmetry becoming more pronounced as drop height increased. This pattern suggests that comparisons based solely on peak values or timing parameters may not fully capture underlying inter-limb imbalances. This is consistent with prior studies reporting absolute symmetry index values exceeding 10% in impact variables, particularly loading rate, which is a sensitive indicator of how rapidly the peak vGRF is absorbed by the body, reflecting the rate at which mechanical energy is transferred through the lower extremity structures during landing. Higher loading rates indicate a shorter time frame for force attenuation, potentially overwhelming musculoskeletal buffering capacity and increasing the likelihood of injury, particularly in repetitive or high-intensity jump tasks (Puddle and Maulder, 2013). This highlights that asymmetry patterns are sport-specific and should be regularly monitored through single-leg landing assessments to tailor corrective training.

### 4.3 Joint work and contribution

The hip and ankle joints play dominant roles during the entire movement, while the knee joint plays a secondary role. Studies have demonstrated that hip extensors and ankle plantar flexors work vigorously during the concentric phase (Yeow et al., 2011). Although joint power was not subjected to statistical analysis in the present study, visual inspection of the joint work contribution charts revealed that the dominant leg consistently demonstrated a stable redistribution pattern across different drop heights: the hip contribution tended to increase, and the ankle contribution decreased. In contrast, the knee contribution remained relatively stable. This observation aligns with prior research, indicating a possible shift of mechanical demand toward proximal joints to maintain movement efficiency (Waterval et al., 2024; Zhao et al., 2024). This coupling of joint moment and work trends suggests a mechanical pattern consistent with a proximal compensation strategy (Arampatzis et al., 2023; Monteiro et al., 2023).

When viewed in conjunction with our joint moment results, an apparent dominance effect emerged at both the hip and ankle, with significantly greater peak moment observed in the dominant leg. The knee joint, by contrast, did not exhibit statistically significant asymmetry (Yeow et al., 2011). This coupling of joint moment and joint work trends reinforces the practical manifestation of a proximal compensation strategy during single-leg drop jump tasks. It also highlights the increasing mechanical demands on the hip during impact absorption and force generation phases. Such joint-level functional asymmetry reveals the differential roles of each joint in unilateral landing strategies. It underscores the need for systematic monitoring and targeted interventions to optimise performance and mitigate injury risk.

Notably, knee joint work remained essentially unchanged at both 30 cm and 40 cm drop heights. The biarticular coupling of the gastrocnemius and other lower limb muscle groups may explain this stability. Previous research in running has shown that the gastrocnemius facilitates bidirectional energy transfer between the knee and ankle joints (Zhang et al., 2025), potentially allowing the knee to act as a passive conduit during impact absorption, thereby reducing mechanical demands on the joint.

From a training and applied perspective, attention should be paid to the potential risks of overusing the hip in the dominant leg (Campbell et al., 2025). Coaches may wish to integrate targeted distal joint conditioning—such as ankle-focused eccentric loading and stability drills—to enhance ankle contribution, balance joint work distribution, and mitigate injury risk (Aout et al., 2025). Furthermore, similar asymmetry-related compensations have been reported in clinical populations such as chronic ankle instability and patellofemoral pain, indicating that our findings may also inform rehabilitation strategies to restore functional symmetry and reduce pain-related movement inefficiencies (Tajdini et al., 2022; Emamvirdi et al., 2023).

In addition, single-leg jump performance has been shown to sensitively detect residual knee function deficits during return-to-sport evaluation after anterior cruciate ligament (ACL) reconstruction (Kotsifaki et al., 2023). Therefore, the asymmetry metrics used in this study may also serve as practical indicators for tracking functional recovery and guiding individualized return to sport (RTS) progression in athletes.

### 4.4 Limitation

Although this study offers valuable insights into inter-limb asymmetries during single-leg drop jumps, some limitations should be noted. The relatively small, homogeneous sample of male athletes from jumping sports (e.g., high jump and long jump), which may exhibit heterogeneity in their specific training and techniques, and the use of only two moderate drop heights (30 cm and 40 cm) may limit generalizability and sensitivity to subtle effects. Future work with larger samples and studies comparing athletes from different specializations could help identify thresholds where kinetic and symmetry parameters diverge. Moreover, the absence of electromyographic (EMG) and dynamic stability measures restricts the interpretation of neuromuscular activation and post-landing control, which should be addressed in future studies.

## 5 Conclusion

The findings demonstrate that the single-leg drop jump test effectively identifies functional lower limb asymmetries. Test data reveal that the dominant leg consistently exhibits superior mechanical characteristics across different drop heights, including shorter ground contact time, greater jump height, and higher GRF. The observed hip-ankle dominant movement pattern further verifies the existence of proximal compensation mechanisms. Importantly, the magnitude of inter-limb asymmetry showed a tendency to increase with drop height, suggesting that higher eccentric demands may amplify existing imbalances. From a training perspective, these results highlight the importance of leveraging the mechanical advantages of the dominant leg while avoiding overreliance through a balanced regimen of bilateral and unilateral exercises. Such an approach can help mitigate injury risks and promote more symmetrical functional performance.

## Data Availability

The raw data supporting the conclusions of this article will be made available by the authors, without undue reservation.
