# Supplementary material for: Lower-limb asymmetries in jump athletes during single-leg drop jump
Source: Front Physiol. 2025 Nov 13;16:1702161. doi: 10.3389/fphys.2025.1702161 (PMC12658979; doi:10.3389/fphys.2025.1702161)
Supplement: Supplementary file 1 [file DataSheet1.pdf]

Table 1. Descriptive Statistics (Mean ± SD) of All Biomechanical Variables Across Drop Heights and Leg Conditions.

Parameters	30cm		40cm	
	NDL	DL	NDL	DL
Hip flexion angle (°)	30.969±7.120	29.766±7.452	29.749±7.863	29.776±4.118
Knee flexion angle (°)	-57.517±7.310	-56.262±4.191	-55.770±12.249	-55.523±7.704
Ankle dorsiflexion angle (°)	99.117±6.059	98.390±4.554	99.410±6.052	101.159±6.445
Hip ROM (°)	25.411±6.124	24.747±6.354	23.419±8.619	23.105±6.140
Knee ROM (°)	-48.661±1.970	-46.567±2.057	-44.613±2.304	-48.531±1.924
Ankle ROM (°)	46.294±8.185	47.106±5.036	45.243±7.529	48.157±6.175
Peak hip moment (Nm/kg)	-5.749±2.159	-6.664±4.410	-5.334±2.726	-7.080±4.217
Peak knee moment (Nm/kg)	5.464±2.276	6.857±3.653	5.839±2.710	6.481±3.234
Peak ankle moment (Nm/kg)	-3.149±0.679	-3.093±0.942	-2.723±0.690	-3.519±1.090
Peak hip power(W/kg)	2.848±2.180	2.989±1.218	3.345±2.295	3.741±2.477
Peak knee power(W/kg)	0.835±0.553	0.872±0.655	1.588±1.182	1.649±1.176
Peak ankle power(W/kg)	0.700±0.558	0.814±0.784	0.855±0.668	0.954±0.821
Contact time (s)	0.293±0.037	0.278±0.048	0.294±0.046	0.284±0.063
Jump height (cm)	0.183±0.048	0.200±0.061	0.195±0.058	0.196±0.051
RSI (m/s)	0.601±0.254	0.717±0.209	0.685±0.196	0.632±0.253
Peak vGRF (BW)	4.199±0.653	4.407±1.083	3.901±0.862	4.704±1.084
Loading rate (BW/s)	18.310±8.469	24.260±12.095	19.102±6.000	26.437±16.069
Time to peak vGRF (ms)	0.236±0.095	0.194±0.074	0.232±0.065	0.212±0.079

RSI: reactive strength index; Peak vGRF: peak vertical ground reaction force

NDL: Non-dominant leg; DL: Dominant leg
